# Clinical presentations, diagnosis, mortality and prognostic markers of tuberculous meningitis in Vietnamese children: a prospective descriptive study

**DOI:** 10.1186/s12879-016-1923-2

**Published:** 2016-10-18

**Authors:** Nguyen Duc Bang, Maxine Caws, Thai Thanh Truc, Tran Ngoc Duong, Nguyen Huy Dung, Dang Thi Minh Ha, Guy E. Thwaites, Doortje Heemskerk, Joel Tarning, Laura Merson, Pham Van Toi, Jeremy J. Farrar, Marcel Wolbers, Thomas Pouplin, Jeremy N. Day

**Affiliations:** 1 Oxford University Clinical Research Unit, Wellcome Trust Major Overseas Programme, 764 Vo Van Kiet, Quan 5, Ho Chi Minh City, Vietnam; 2 Pham Ngoc Thach Hospital, 120 Hung Vuong, Quan 5, Ho Chi Minh City, Vietnam; 3 Department of Clinical Sciences, Liverpool School of Tropical Medicine, Pembroke Place, L3 5QA Liverpool, UK; 4 Centre for Tropical Medicine and Global Health, Nuffield Department of Medicine Research Building, University of Oxford, Old Road campus, Roosevelt Drive, Oxford, UK; 5 Mahidol-Oxford Tropical Medicine Research Unit, Faculty of Tropical Medicine, 420/6 Ratchawithi Rd., Bangkok, Thailand

**Keywords:** Tuberculous meningitis, Children, Vietnam, Mycobacterium, Survival

## Abstract

**Background:**

Tuberculous meningitis in adults is well characterized in Vietnam, but there are no data on the disease in children. We present a prospective descriptive study of Vietnamese children with TBM to define the presentation, course and characteristics associated with poor outcome.

**Methods:**

A prospective descriptive study of 100 consecutively admitted children with TBM at Pham Ngoc Thach Hospital, Ho Chi Minh City. Cox and logistic regression were used to identify factors associated with risk of death and a combined endpoint of death or disability at treatment completion.

**Results:**

The study enrolled from October 2009 to March 2011. Median age was 32.5 months; sex distribution was equal. Median duration of symptoms was 18.5 days and time from admission to treatment initiation was 11 days. Fifteen of 100 children died, 4 were lost to follow-up, and 27/81 (33 %) of survivors had intermediate or severe disability upon treatment completion. Microbiological confirmation of disease was made in 6 %. Baseline characteristics associated with death included convulsions (HR 3.46, 95CI 1.19–10.13, *p* = 0.02), decreased consciousness (HR 22.9, 95CI 3.01–174.3, *p* < 0.001), focal neurological deficits (HR 15.7, 95CI 1.67–2075, *p* = 0.01), Blantyre Coma Score (HR 3.75, 95CI 0.99–14.2, *p* < 0.001) and CSF protein, lactate and glucose levels. Neck stiffness, MRC grade (children aged >5 years) and hydrocephalus were also associated with the combined endpoint of death or disability.

**Conclusions:**

Tuberculous meningitis in Vietnamese children has significant mortality and morbidity. There is significant delay in diagnosis; interventions that increase the speed of diagnosis and treatment initiation are likely to improve outcomes.

## Background

Tuberculosis (TB) is an important cause of childhood morbidity - recent estimates of the number of children developing TB each year range from 500 000 to 1 million, significantly higher than previously estimated by the WHO [1, 2]. There may be 50 million children latently infected [3]. 5 % of disease occurs in HIV co-infected children, although this is higher in sub-Saharan Africa [3]. Despite this high burden, in TB as in many other diseases, children are a neglected group - the vast majority of data used to guide management are derived from adult patients.

Tuberculous meningitis (TBM) is the most severe form of tuberculosis, with high rates of disability and death [4]. Children are more likely than adults to develop disseminated TB and TB meningitis following infection [5]. This risk is greatest for infants and children under 2 years of age, probably due to the immaturity of the immune system [6]. The challenges of TBM diagnosis and management are exacerbated in children. Initial symptoms in infants are non-specific, it is difficult to obtain diagnostic samples and more invasive sampling methods are needed, and the diagnostics samples obtained generally proffer extremely low yields of mycobacteria [7, 8]. Death from TBM is strongly associated with delays in diagnosis and treatment both in children and adults [9–12]. It is striking that there has never been a randomized controlled trial of anti-tuberculosis drugs in children with TBM [13].

Vietnam is classified as having a high burden of TB, with a prevalence in the order of 145 per 100 000 of the population as a whole [14]. Tuberculous meningitis in adults is well characterized in Vietnam, and these data have been key in developing treatment guidelines, but there are no data on the disease in children [11, 15–17]. In accordance with historic WHO policy, the Vietnamese National TB Programme has focused on the systematic notification of smear positive cases of TB. However, the majority of pediatric TB cases are smear negative, and subsequently limited data are available on pediatric TB in Vietnam. In line with WHO policy revisions, Vietnam has recently implemented disaggregated data collection for pediatric TB. Improving outcomes in children is dependent upon a better understanding of the epidemiology of childhood TB, and early diagnosis and instigation of effective treatment.

## Methods

### Study aim

This study aimed to describe the current practice in the management of Vietnamese children with TBM, the presenting clinical features and predictors of poor outcome (death and neurological disability), in order to define the challenges in management and prioritise research.

### Study design

A prospective descriptive study of 100 consecutive cases of TBM in children (aged ≤15 years).

### Setting and participants

The study was conducted at Pham Ngoc Thach Hospital (PNT), Ho Chi Minh City (HCMC). PNT is the tertiary referral centre for tuberculosis in the south of Vietnam, serving 40 million people. All children diagnosed or suspected of having TBM in HCMC and the surrounding provinces are referred here. Approximately 500 inpatient cases of pediatric TB are treated each year; around 20 % have meningitis.

### Entry criteria

All patients aged ≤15 years presenting to Pham Ngoc Thach Hospital with a clinical syndrome consistent with TBM (meaning one or more of: fever, headache, neck stiffness, vomiting, confusion, coma, convulsions, cranial nerve palsies, hemiplegia or paraplegia) and considered to have tuberculous meningitis by their attending physician (i.e. meriting anti-TB therapy) were eligible to enter the study. TBM was classified as “definite”, “probable” or “possible”, as follows:

- Definite TBM: acid-fast bacilli (AFB) seen or cultured from cerebrospinal fluid (CSF).
- Probable TBM: Clinical syndrome consistent with TBM, and one or more of the following criteria: suspected active pulmonary tuberculosis on chest radiography, AFB found in any specimen other than the CSF, brain imaging consistent with TBM, or clinical evidence of other extrapulmonary tuberculosis.
- Possible TBM: Clinical syndrome consistent with TBM and at least 4 of the following: past medical history of tuberculosis, predominance of lymphocytes in the CSF, illness of ≥6 days in duration, CSF: blood glucose ratio <0.5, altered consciousness, yellow CSF, and focal neurological signs.

### Patient assessment

All patients had clinical assessments by the dedicated study team at study entry, 2 weeks, 1, 2, 3 and 8 months.

### Lumbar puncture

All patients underwent lumbar puncture either at the referring centre or on admission to PNT hospital. Lumbar puncture was repeated at days 30 and 90 following study entry/treatment initiation. Cerebrospinal fluid (CSF) investigations included cell count, glucose, protein, lactate, Gram’s stain, and India ink test, and culture for fungi and pyogenic bacteria. CSF Ziehl-Neelsen smear and culture were not available at referring centres. When patients were referred from other centres, lumbar puncture was only repeated at baseline if felt to be clinically indicated by the attending physician. The GeneXpert test was not available at the time the study took place.

### Other investigations

All children had haematology and biochemistry investigations according to standard of care in the hospital. Sputum was examined for acid fast bacteria (AFB) when available; gastric washings for AFB were done at the discretion of the attending physician. All patients had HIV testing - counselling and HIV testing were available for the parents of children diagnosed with HIV. All patients had a chest radiograph performed at study entry. 40 patients were entered into a descriptive radiological sub-study and underwent cranial Magnetic Resonance Imaging (MRI) at study entry, on day 60 and day 270. Other imaging was performed at the discretion of the attending physician.

### Classification of severity

All patients were graded for severity at study entry. For children older than 5 years severity was graded according to a modified United Kingdom Medical Research Council criteria based on Glasgow Coma Score: Grade I had a Glasgow coma score (GCS) of 15/15 with no focal neurological signs, grade II either had a GCS 11–14 or GCS 15 with focal neurological signs, Grade III had a GCS of ≤ 10.

For children less than 5 years of age severity of TBM was graded according to the Blantyre Coma Score. Patients with grade I disease had a Blantyre coma score of 4–5 with no focal neurological signs; patients with grade II disease had a Blantyre coma score of 2–3 or a score of 4–5 with focal neurological signs; and grade III had a Blantyre coma score ≤1.

### Treatment and follow-up

Anti-tuberculosis treatment was according to Vietnamese treatment guidelines, which at the time were consistent with the 2006 WHO guidelines. Patients received oral isoniazid (5 mg/kg), rifampicin (10 mg/kg), pyrazinamide (25 mg/kg), ethambutol 15 mg/kg and intramuscular streptomycin (15 mg/kg) for 2 months, followed by oral isoniazid, rifampicin, pyrazinamide and ethambutol for 1 month and oral isoniazid, rifampicin, and ethambutol at the same doses for 5 months [2HRZES/1HRZE/5HRE]. Streptomycin was not used for HIV-infected patients. Patients with MRC grades II and III and BCS grades II and III disease received adjuvant dexamethasone for the first six weeks; patients with MRC grade I and BCS grade I received adjuvant dexamethasone for 4 weeks as per Vietnamese guidelines [11]. All patients were reviewed daily by the study team while in-patients, at days 30, 60 and 90, and on completion of treatment after 8 months. DOT was used to secure and monitor treatment adherence, and drugs were administered via nasogastric tube to unconscious children.

### Assessment of outcome

We were interested in two outcomes: survival, and a combined endpoint of neurological disability or death. Disability status was assessed at the end of 8 months of treatment with the use of the two simple questions (“Does the child require help from anybody for everyday activities [e.g., eating, drinking, washing, brushing teeth, and going to the toilet]?” and “Has the illness left the child with any other problems?”) and the modified Rankin scale (scores range from 0 [no symptoms at all] to 6 [death]) and was classified as good (i.e., no disability), intermediate, severe, or death, as described elsewhere [11]. For children under 5 years, the response was determined by the study clinician in relation to the expected functioning in normal children of the same age.

### Study size

The study was designed as a prospective descriptive study to include all patients less than or equal to 15 years of age presenting with suspected TBM to Pham Ngoc Thach Hospital. Approximately 100 such patients are admitted each year, and we planned to recruit 100 consecutively admitted patients.

### Statistical analysis

The raw data was imported into a secure anonymised in-house data management system (CliRes). Between group comparisons of baseline characteristics were based on the Wilcoxon rank sum test and Fisher’s exact test for continuous and categorical variables, respectively. Mortality at 8 months was estimated with the Kaplan-Meier method. Univariate Cox regression was used to identify significant factors influencing time to death. Factors relating to the probability of a combined endpoint of neurological sequelae (intermediate or severe disability) or death were determined using univariate logistic regression and the Firth correction was applied in case of separation [18]. Multivariable regression models were not fitted due to the relatively low number of events and large number of factors of interest. All analyses were performed using R software version 2.9.1 [19].

## Results

### Demographic and clinical characteristics

Enrollment was conducted between October 2009 and March 2011. During this period 133 children were admitted to PNT hospital with suspected TBM. Twelve children declined to join the study and 21 were not recruited because they were already receiving treatment. Follow-up completed in December 2011. The median duration from first hospital admission to making the diagnosis of TBM (and instigation of treatment) was 11 days (range 1–74 days).

Baseline characteristics and outcomes for the cohort are shown in Table 1.

**Table 1:** Baseline characteristics and outcomes of 100 childhood TBM patients

Symptoms and signs	Number affected (%)		Median duration in days (range)
Fever	96/97 (99 %)		23; (6–96)
Vomiting	76/100 (76 %)		13; (1–96)
Weight loss	59/98 (60 %)		15.5; (7–90)
Confusion	15/100 (15 %)		10.4; (2–30)
Unconsciousness	27/100 (27 %)		5.3; (1–16)
Headache^a^	49/55 (89 %)		21; (4–66)
Fits	39/100 (39 %)		8.5; (1–35)
Neck stiffness	75/100 (75 %)		
Hemiparesis/Hemiplegia	20/98 (20 %)		
Paraparesis/paraplegia	6/98 (6 %)		
Monoplegia	4/98 (4 %)		
Quadriplegia	2/98 (2 %)		
II nerve involvement	3/98 (3 %)		
III nerve palsy	4/98 (4 %)		
VI nerve palsy	20/98 (20 %)		
VII nerve palsy	7/98 (7 %)		
Anemia (Hb < 11 g/dL)	46/99 (46 %)		
HIV infection	4/96 (4 %)		
HBV infection	5/96 (5 %)		
MRC Grades >5 years (33)	I: 16 (48 %)	II: 11 (33 %)	III: 6 (18 %)
Blantyre < 5 years (67)	4–5: 43 (64 %)	2–3: 12 (18 %)	0–1: 12 (18 %)
Deaths	15/100 (15 %)		
Sequelae			
Severe	6/81 (7 %)		
Intermediate	21/81 (26 %)		

^a^Unable to ascertain in 44 due to the age of the child; headache was not assessed for one additional subject

Fifty-six percent (*n* = 56/100) of patients were male. The majority of children (67 %) were less than 5 years old (median age 32.5 months, range 2 to 180 months). Four of 96 children tested were HIV infected (4 %); 5 % (*n* = 5/96) were hepatitis B surface antigen positive. Concomitant extra-pulmonary TB was common, seen in 42 patients (36 pulmonary, 4 pulmonary with peripheral lymphadenitis, 1 pulmonary disease with pleural involvement, and 1 pulmonary disease with concomitant TB arthritis. A family history of TB was reported for 27 patients (27 %); one patient had a previous history of TB.

Fever, vomiting and nuchal rigidity were the most frequently elicited symptoms. Seizures were common reported in 39 cases. Headaches occurred in at least 49 (49 %) cases - in 44 cases (44 %) the presence of headache was uncertain because the child was too young to describe the symptom. Of 33 (33 %) children ≥ 5 years of age, 16 (48 %) had MRC grade I disease, 11 (33 %) grade II disease, and 6 (18 %) grade III disease. The remaining 67 children under 5 years were graded using the Blantyre coma score (BCS): BCS I (4–5): 43 (64 %), BCS II (2–3): 12 (18 %) and BCS III (0–1): 12 (18 %) (Table 1). Focal neurological signs were frequent occurring in 58 %, most commonly VIth cranial nerve palsies (22 %), followed by hemiplegia (20 %), VIIth cranial nerve palsy 7 %, paraplegia 6 %, monoparesis 4 %, IIIrd cranial nerve palsy 4 %, optic nerve atrophy 3 %, and quadriparesis 2 %. Four patients (4 %) were lost to follow up after 3 months of treatment.

### Cerebrospinal fluid results

Lumbar puncture was performed on all patients (*n* = 100) at referring hospitals; 22 (22 %) children had repeat lumbar puncture at PNT. All CSF was analyzed for protein, lactate and glucose concentrations, and for cell count and differentials (Table 2). The median white cell count was 202 cells/uL. 92 of 95 (97 %) patients had lymphocyte predominance (>50 %). The mean CSF:blood glucose ratio was 0.25 (range 0.07 to 0.69; 7 of 100 patients had ratios >0.5). 77 patients had CSF lactate measured; the median was 5.3 mmol/L (10th and 90^th^ centiles 2.36 and 8.8 mmol/L respectively). All but 8 patients had elevated CSF protein concentrations (median 1.2 g/L, upper limit of the normal range 0.4 g/L).

**Table 2:** Baseline characteristics of possible TBM and definite/probable TBM

Variables	Possible TBM (*n* = 28)	Definite/Probable TBM (*n* = 72)	*P*-value for comparison
Age^a^ (months)	24.5 (2–165)	36.5 (2–180)	0.09
Male (%)	18/28 (64 %)	38/72 (53 %)	0.03
Fever	28/28 (100 %)	71/72 (99 %)	0.99
Symptom duration^a^ (days)	16 (6–60)	19.5 (6–96)	0.172
Vomiting	20/28 (71 %)	56/72 (78 %)	0.50
Altered sensorium	6/28 (21 %)	36/72 (50 %)	0.01
Fits	9/28 (32 %)	30/72 (40 %)	0.38
Neck stiffness	17/28 (61 %)	58/72 (81 %)	0.04
Motor deficit	4/25 (16 %)	28/60 (47 %)	0.01
Cranial nerve palsy	9/28 (32 %)	18/72 (25 %)	0.91
HIV infection	1/28 (4 %)	3/72 (4 %)	0.99
HBV infection	2/28 (7 %)	3/72 (4 %)	0.86
Family TB history	7/28 (25 %)	19/72 (26 %)	0.89
Death	2/27 ^b^ (7 %)	13/69 (19 %) ^c^	0.28
Permanent sequelae	2/25 (8 %)	25/56 (45 %)	0.002
CSF WCC^a^ (cells/uL)	144 (4–780)	150 (1–802)	0.37
CSF Lymph^a^ (cells/uL)	80 (40–100)	80 % (50–100)	0.44
CSF Protein^a^ (g/L)	0.8 (0.2–5)	1.3 (0.1–4.8)	0.08
CSF Lactate^a^ (mmol/L)	4.3 (1.4–8.8)	5.6 (1.21–19.2)	0.08
CSF: Blood Glucose ratio^a^	0.30 (0.09–0.61)	0.25 (0.07–0.69)	0.51

^a^ Values are medians/ranges

^b^1 lost to follow-up

^c^3 lost to follow-up

### Microbiology results

None of 22 CSF samples taken at PNT were AFB smear positive, However, *M. tuberculosis* was cultured from 6 of these samples. Smears of sputum and gastric aspirates were positive for AFB in only 2 (7 %) of 29 patients and 5 (7 %) of 67 patients, respectively. Drug susceptibility testing was not performed.

### TBM classification

Six cases met the definition of definite TBM. Sixty-six cases fulfilled the case definition of probable TBM: seven had positive AFB smears and/or *M. tuberculosis* cultured from sputum or gastric aspirate; 33 had abnormal chest X-rays consistent with TB and an abnormal brain imaging, and 26 cases had an abnormal CT or MRI brain imaging consistent with TBM.

The remaining 28 cases fulfilled the definition of possible TBM, with clinical features and CSF biochemistry consistent with the diagnosis. The differences in baseline characteristics and outcome between definite/probable and possible cases are illustrated in Table 2. There were statistically significant lower rates of altered sensorium, neck stiffness and motor deficits at baseline, and a reduced risk of neurological sequelae in possible cases, suggesting that these patients may have had less severe disease.

### Imaging findings

All children had chest X-ray examinations at admission: 42 % (42/100) had abnormalities consistent with TB (14 % mediastinal lymphadenopathy 14 %, consolidation 11 %, miliary pattern 9 %, nodules 6 %, cavity 1 % and atelectasis 1 %).

Forty-three patients underwent cranial MRI before beginning treatment (42 with contrast enhancement). Findings are shown in Table 3.

**Table 3:** Baseline brain MRI scan results for 43 patients

Finding	Absolute frequency (%)
Any abnormality	37/43 (86 %)
Meningeal enhancement	26/42 (62 %)
Basal	25 (60 %)
Sylvian Fissure	16 (37 %)
Suprasellar cistern	4 (9 %)
Hydrocephalus	19/43 (44 %)
Tuberculoma	6/42 (14 %)
Infarction	13/43 (30 %)
Basal Ganglia	8/43 (19 %)
Cerebral cortex	3/43 (7 %)
Internal capsule	1/43 (2 %)
Brain stem	1/43 (2 %)

Abnormalities were frequent, detected in 86 % of patients: 62 % (26/42) had basal meningeal enhancement, 44 % (19/43) hydrocephalus, 30 % (13/43) infarctions and 14 % (6/42) tuberculomas. 35 of these 43 patients had repeat cranial MRI scans performed 60 days post randomization: 37 % (13/35) had basal meningeal enhancement, 29 % (10/35) hydrocephalus, 34 % (12/35) infarctions and 29 % (10/35) tuberculomas.

### Death and sequelae

Fifteen patients died by 8 months (Kaplan-Meier estimate of mortality 15.7 %, 95CI: 9.56–24.3 %, 4 patients lost to follow-up). Eight (53 %) deaths occurred within the first 6 days of treatment; 14 within 45 days of diagnosis. At the end of treatment, six of 81 surviving patients (7.4 %) were classified with severe disability and 21/81 (26 %) were classified with intermediate disability (Table 1).

Baseline predictors associated with increased risk of death or neurological sequelae are shown in Table 4.

**Table 4:** Univariate analysis of potential predictors of mortality and neurological sequelae in patients with tuberculous meningitis

Characteristic	Time to death (*N* = 100)			Neurological sequelae or death (*N* = 96)		
	Death/No at risk	HR (95 % CI)	*P*	Events/No at risk	OR (95 % CI)	*P*
Sex						
^a^Male	10/56 (17.9 %)	0.61	0.36	23/54 (42.6 %)	1.11	0.80
Female	5/44 (11.4 %)	(0.21–1.78)		19/42 (45.2 %)	(0.49–2.52)	
Age						
^a^<5 years	11/67 (16.5 %)	0.70	0.54	30/64 (46.9 %)	0.68	0.38
≥ 5 years	4/33 (12.1 %)	(0.22–2.20)		12/32 (37.5 %)	(0.28–1.61)	
Duration of symptoms *N* = 89		1.00	0.4	–	1.01	0.43
(per +1 day)		(0.98–1.01)			(0.98–1.04)	
Neck stiffness						
^a^No	1/25 (4.0 %)	5.15	0.11	5/24 (20.8 %)	4.02	0.01
Yes	14/75 (18.7 %)	(0.68–39.16)		37/72 (51.4 %)	(1.44–13.18)	
HIV co-infection						
^a^No	13/92 (14.1 %)	1.85	0.55	39/88 (44.3 %)	0.42	0.43
Yes	1/4 (25.0 %)	(0.24–14.17)		1/4 (25.0 %)	(0.02–3.42)	
BCS Grade						
age <5 years						
^a^BI	0/43 (0 %)	1.00	<0.001	10/40 (25.0 %)	1.00	<0.001
BII	3/12 (25.0 %)	27.96 ^b^		9/12 (75.0 %)	9.00	
		(2.71–3760)			(2.21–47.06)	
BIII	8/12 (67.0 %)	94.64 ^b^		11/12 (91.7 %)	33.00	
		(11.71–12,260)			(5.44–643.12)	
TBM grade						
(age ≥ 5 years)						
^a^I	1/16 (6.3 %)	1.00	0.28	2/15 (13.3 %)	1.00	0.01
II	1/11 (9.1 %)	1.41		5/11 (45.5 %)	5.41	
		(0.09–22.5)			(0.81–36)	
III	2/6 (33.3 %)	6.26		5/6 (83.3 %)	32.5	
		(0.57–69.2)			(2.4–443)	
Convulsions						
^a^No	5/61 (8.2 %)	3.46	0.02	20/59 (33.9 %)	2.86	0.01
Yes	10/39 (25.6 %)	(1.18–10.13)		22/37 (59.5 %)	(1.24–6.81)	
Decreased level of consciousness						
^a^No	1/58 (1.7 %)	22.9	<0.001	10/54 (18.5 %)	14.08	<0.001
Yes	14/42 (33.3 %)	(3.01–174.3)		32/42 (76.2 %)	(5.46–39.85)	
Focal neurological deficits						
^a^No	0/53 (0 %)	15.66	0.01	6/50 (12.0 %)	15.4	<0.001
Yes	4/32 (12.5 %)	(1.67–2075)		21/31 (67.7 %)	(5.22–51.96)	
Extra meningeal TB						
^a^No	6/51 (11.8 %)	2.3	0.11	19/57 (33.3 %)	2.87	0.01
Yes	9/30 (30.0 %)	(0.82–6.45)		23/39 (59.0 %)	(1.25–6.80)	
TB culture						
^a^Negative	2/14 (14.3 %)	2.72	0.33	4/16 (25.0 %)	4.50	0.16
Positive	2/3 (66.7 %)	(0.38–19.4)		3/5 (60.0 %)	(0.56–45.4)	
Hydrocephalus^c^						
^a^No	2/31 (6.5 %)	3.46	0.10	6/31 (19.4 %)	9.72	<0.001
Yes	6/30 (20.0 %)	(0.70–17.16)		21/30 (70.0 %)	(2.97–32)	
Cranial nerve palsy						
No	14/70 (20.0 %)	5.42	0.10	29/70 (41.4 %)	0.71	0.45
Yes	1/26 (3.8 %)	(0.71–42.2)		13/26 (50.0 %)	(0.29–1.75)	
CSF Protein	*N* = 99	1.74	0.002	*N* = 94	1.65	0.01
(per + 1 g/L)		(1.22–2.49)			(1.14–2.51)	
CSF Lactate	*N* = 77	1.14	0.04	*N* = 73	1.20	0.03
(per + 1 mmol/L)		(1.01–1.29)			(1.01–1.47)	
CSF Glucose	*N* = 100	0.45	0.05	*N* = 96	0.65	0.10
(per + 1 mmol/L)		(0.20–0.98)			(0.38–1.08)	

^a^Baseline category. HR and OR give comparisons to the baseline category. *P*-value is an overall likelihood ratio test for the significance of the factor

^b^Model fitted by logistic regression with Firth correction

^c^hydrocephalus diagnosed by MRI brain (43 patients or CT brain 19 patients)

More severe BCS was predictive of increased risk of death, as were history of coma, fits, focal neurological deficits and CSF levels of protein, lactate and glucose. The study did not detect a statistically significant association between risk of death and MRC score but this may be due to the low number of children in the study aged ≥5 years. However, there was a consistent increase in the hazard of death as MRC grade worsened. Hydrocephalus, neck stiffness and MRC grade at baseline were associated with an increased risk of the combined endpoint of neurological sequelae or death (*p* = 0.01). Neither brain infarctions nor basal meningeal enhancement identified on brain imaging were associated with mortality or the combined endpoint.

Two patients developed drug induced liver injury (DILI) according to the classification of antituberculosis drug-induced hepatotoxicity based on the WHO Adverse Drug Reaction Terminology [20]. This occurred at 2 and 4 weeks following treatment initiation. Neither case was infected with Hepatitis B or C virus. In both cases, PZA was discontinued completely and INH and RIF were reintroduced successfully. Both patients survived.

### Uniform case definition for tuberculous meningitis

After this study was designed and recruited, a consensus document was published suggesting criteria to standardize the categorization of TB meningitis as possible, probable or definite, in order to allow comparison of different populations in intervention trials [21]. The criteria are based upon the expert opinion of a number of international experts. We reclassified our patients according to these new criteria, and this resulted in 18 cases moving from the ‘probable’ diagnosis to the new ‘possible’ category, and 8 cases moving from our ‘possible’ category to the new ‘probable’ category. Following re-categoristaion, male sex was no longer statistically significantly differently distributed between possible and probable/definite cases; altered sensorium, neck stiffness, motor deficit and permanent sequelae remained significantly more common amongst probable and definite cases. Cranial nerve palsies and death became significantly more associated with probable and definite cases.

## Discussion

This study demonstrates the challenges in delivering effective treatment for TBM in children in Vietnam. First, TBM is associated with considerable mortality and morbidity, with a risk of death at the end of treatment of 15 %, and 33 % of survivors having intermediate or severe disability. These figures are similar to those reported from other studies in children, and to the rates of death and disability seen in HIV uninfected adults with TBM in Vietnam, but while in adult disease considerable progress has been made and large studies are on-going, childhood TBM remains a neglected area [11, 13, 22–29]. Treatment guidelines for children are largely derived from studies in adults, and although efforts are underway to develop pediatric formulations in appropriate ratios, at the present time dosing schedules are limited by adult fixed dose combination tablets. Randomised controlled trials and pharmacokinetic studies of novel dosing strategies and agents in children are needed to ensure treatment is optimized.

Secondly, the timely instigation of treatment in TBM remains a significant challenge, even in countries such as Vietnam where the burden of TB is high and the diagnosis is at the forefront of clinicians’ minds. In this study, the median time from presentation to treatment was 19 days, and 50 % of deaths occurred within the first 6 days of treatment. Earlier instigation of treatment may be able to prevent these deaths. Delays in diagnosis and treatment are likely explained by 3 main factors. First, poverty is a barrier to accessing health care. Since the study Vietnam has made important progress in rolling out free access to healthcare for children under the age of 6 years; however, this is beyond the means of many countries where the TB burden is highest. Secondly, TBM presents in a non-specific manner and it is rare to confirm the diagnosis microbiologically even in high resource settings. In this study, a definitive diagnosis was made in only 6 % of cases. This is partly explained because only 22 % (22/100) of patients had a CSF smear and culture, although the study is representative of real world practice. Of note, no patients were CSF smear positive. Most papers report finding AFB in fewer than 20 % of TBM patients [30]. Improving laboratory training and infrastructure, and improving access to WHO endorsed technologies such as the GeneXpert, would potentially reduce this delay but will not alter the fact that clinical specimens from children are limited both by their volume and their paucibacillary nature [31]. An adequate volume of CSF significantly affects the likelihood of confirming the diagnosis [32] and appropriate sampling for TB at other sites should be performed. Moreover, while TB diagnostic tests remain poorly sensitive, increasing the capacity to identify and exclude other central nervous system pathogens is key in giving clinicians the confidence to instigate treatment in a timely manner and will reduce unnecessary prescription of lengthy TB regimens. Of note, an approach that has shown promise in South African children with TBM, which resulted in a doubling of diagnostic sensitivity, is the use of more than one nucleic acid amplification test on CSF [33]. However, the investigators still found a combination of microscopy and culture to be most effective. We have found GeneXPert and CSF smear to have similar sensitivities of in adult patients; the challenge for Vietnam remains in obtaining significant volumes of CSF from infants and balancing the risk of investigation with the benefits of a definitive diagnosis [31]. The early identification of drug resistance is likely to be an important factor in improving outcomes. A South African study found that drug resistance was associated with delays in instigation of effective treatment, and that multidrug resistance was associated with worse outcomes [34]. In this study isoniazid mono-resistance was not clearly associated with worse outcomes [34]. However, this mimics the experience in adult patients – initial small studies failed to show a deleterious effect of isoniazid mono-resistance on outcome, but later larger datasets demonstrated that indeed such resistance is disadvantageous [35, 36].

Thirdly, TB treatment is onerous, both in regards to side effects and duration, which may lead to reluctance to start empirical treatment where the risk and consequences of disease are perceived to be low. However, *M. tuberculosis* has been reported to be a more frequent cause of meningitis than pyogenic organisms in South Africa – given that 50 % of deaths are occurring within the first week of treatment a case could be made for early empirical TB treatment in such centres [37]. Few data exist on the optimal duration of treatment for TBM. Drugs such as the fluoroquinolones, which have good tolerability and excellent CSF penetration, potentially offer more effective and palatable treatment regimens, but are relatively untested in children [38]. Randomized controlled trials in children specifically designed to improve TB treatment by 1) identifying shorter equally effective courses and 2) new treatment combinations, may go some way to addressing this issue, although recent trials in adult patients have had mixed results [39–42]. The effective dose of rifampicin appears to be key in improving outcomes. Such regimes would enable a move towards an empirical therapeutic approach similar to that used in acute bacterial meningitis to be employed in high burden settings.

Finally, it is reassuring that drug induced liver injury necessitating treatment interruption was rare and not associated with a poor outcome in this study, affecting just 2 % of the patients. Our study is small and thus our estimates are somewhat imprecise. However, the rate is considerably lower than the 13 % quoted for DILI in adults, supporting the concept that higher doses could be used in children, as advocated in the recent WHO guidelines. [2, 43–45].

Our study had some limitations. It was limited by its small size, and thus estimates around the rates of death and other sequelae lack precision, although findings are generally in keeping with other series [13]. Secondly, we used all-cause mortality. However, 50 % of all deaths occurred within 1 week of treatment initiation, and the rates of co-morbidities (such as HIV) were low, meaning that it is reasonable to assume that all deaths were either a direct consequence of TB or a result of its sequelae. Thirdly, children were only followed up until the end of treatment, and we may have missed later deaths and relapse. The main limitation is the low rate of microbiological confirmation of tuberculosis. This is consistent with other studies in children and represents the very real practical difficulties of managing these children. It is possible that some of these cases, particularly those meeting the ‘possible’ case definition, may have had some other pathology. However, when the possible cases are excluded, then the mortality and morbidity rate rises further underlying the need to improve diagnostics and treatment for this devastating disease.

Our study was designed and completed before the publication of the universal case definition (UCD) for TBM was published and thus we present our results as originally intended, with a secondary analysis using this classification [21]. The UCD is based upon a non-linear scoring system derived from expert opinion, and has been shown to misclassify 14 % of cases of culture proven TBM as possible TBM in children in a South African study [46]. This underlines the danger of using this research tool as a diagnostic aid, particularly where microbiological diagnostic facilities are poorly developed. Moreover, it is not yet clear that the UCD achieves that which it set out to achieve – i.e allowing robust comparison of patients across diverse study groups according to the UCD defined likelihood of there being definite, probable or possible TBM. As an illustration, a study from South Africa found that patients with ‘possible’ versus other ‘probable/definite’ TBM had significant differences in CSF protein concentrations and CSF/serum glucose concentrations. This difference was not apparent in our children, suggesting that our ‘possible’ cases may be more likely to actually have TBM than possible cases from South African series [47]. There is a need to develop more robust data-driven classification systems, and given the number of patients enrolled into intervention trials in the last 15 years this should be feasible [11, 15, 38, 48].

## Conclusions

Tuberculous meningitis in children carries significant morbidity and mortality in Vietnam. A particular challenge is in reducing the time to diagnosis, and institution of effective treatment. However, improving outcomes will also require the development of treatment regimens tailored to children. Given the recent disappointing results of fluoroquinolone boosted treatment regimens in adults with TBM, randomized controlled trials of boosted rifampicin dosing would seem the most important strategy to develop.

## Abbreviations

AFB: Acid fast bacilli
BCS: Blantyre coma score
CSF: Cerebrospinal fluid
DILI: Drug induced liver injury
DOT: Directly observed treatment
E: Ethambutol
GCS: Glasgow coma score
H: Isoniazid
HCMC: Ho Chi Minh City
HIV: Human immunodeficiency virus
INH: Isoniazid
MRC: Medical Research Council
MRI: Magnetic Resonance Imaging
PNT: Pham Ngoc Thach Hospital
PZA: Pyrazinamide
R: Rifampicin
RIF: Rifampicin
S: Streptomycin
TB: Tuberculosis
TB: Tuberculosis
TBM: Tuberculous meningitis
UK: United Kingdom
WHO: Word Health Organisation
Z: Isoniazid
